# Graft-Specific Regulatory T Cells for Long-Lasting, Local Tolerance Induction

**DOI:** 10.3390/cells13141216

**Published:** 2024-07-19

**Authors:** Nadja Seltrecht, Matthias Hardtke-Wolenski, Konstantinos Iordanidis, Danny Jonigk, Melanie Galla, Axel Schambach, Laura Elisa Buitrago-Molina, Heiner Wedemeyer, Fatih Noyan, Elmar Jaeckel

**Affiliations:** 1 Department of Gastroenterology, Hepatology, Infectious Diseases & Endocrinology, Hannover Medical School, 30625 Hannover, Germanybuitrago.laura@mh-hannover.de (L.E.B.-M.); wedemeyer.heiner@mh-hannover.de (H.W.); elmar.jaeckel@uhn.ca (E.J.); 2 Institute of Medical Microbiology, University Hospital Essen, University of Duisburg-Essen, 45147 Essen, Germany; 3 Institute of Pathology, Hannover Medical School, 30625 Hannover, Germany; 4 Institute of Experimental Haematology, Hannover Medical School, 30625 Hannover, Germany; galla.melanie@mh-hannover.de (M.G.); schambach.axel@mh-hannover.de (A.S.); 5 Department of Liver Transplantation, Multi Organ Transplant Program, Toronto General Hospital, United Health Network, University of Toronto, Toronto, ON M5G 2N2, Canada

**Keywords:** solid organ transplantation, regulatory T cells, alloantigen-specific, immune tolerance

## Abstract

Background: Solid organ transplantation is hindered by immune-mediated chronic graft dysfunction and the side effects of immunosuppressive therapy. Regulatory T cells (Tregs) are crucial for modulating immune responses post-transplantation; however, the transfer of polyspecific Tregs alone is insufficient to induce allotolerance in rodent models. Methods: To enhance the efficacy of adoptive Treg therapy, we investigated different immune interventions in the recipients. By utilizing an immunogenic skin transplant model and existing transplantation medicine reagents, we facilitated the clinical translation of our findings. Specifically, antigen-specific Tregs were used. Results: Our study demonstrated that combining the available induction therapies with drug-induced T-cell proliferation due to lymphopenia effectively increased the Treg/T effector ratios. This results in significant Treg accumulation within the graft, leading to long-term tolerance after the transfer of antigen-specific Tregs. Importantly, all the animals achieved operational tolerance, which boosted the presence of adoptively transferred Tregs within the graft. Conclusions: This protocol offers a means to establish tolerance by utilizing antigen-specific Tregs. These results have promising implications for future trials involving adoptive Treg therapy in organ transplantation.

## 1. Introduction

Chronic graft dysfunction after solid organ transplantation (SOT) remains a major clinical concern. In addition, the side effects of chronic immunosuppression, such as loss of kidney function, infections, and cancer, contribute to morbidity after transplantation [1,2,3]. Although operational tolerance has been achieved in selected cases after liver and kidney transplantation [4,5,6], most patients will require lifelong immunosuppressive therapy [7,8]. Regulatory T cells (Tregs) are key regulators of immune homeostasis and are involved in dominant self-tolerance maintenance [9,10,11]. They are characterized by the constitutive expression of Foxp3.

Immunomodulatory Tregs are a promising tool for interventional therapy after SOT because they can achieve general immune competence and prevent graft rejection. Tregs play a pivotal role in controlling alloimmune responses after transplantation [12], as depletion of Tregs leads to rapid rejection [13]. However, adoptive transfer of polyspecific Tregs was unable to control transplant rejection under non-lymphopenic conditions [14]. Polyclonal nTregs as a therapy for the prevention of graft-versus-host disease (GvHD) are being studied in clinical trials (ClinicalTrials.gov, NCT03683498, and NCT01911039).

Tang et al. showed that tolerance induction by adoptive Treg transfer requires depletion of allospecific T effector cells (Teffs) by the application of cyclophosphamide after transplantation [15]. In line with these results, Todo et al. conducted an important proof-of-concept weaning study in patients receiving left-lobe liver transplantation. The researchers successfully combined post-transplant cyclophosphamide with the transfer of enriched Treg cells before weaning patients six months after transplantation [16]. Nonetheless, for SOT, the systemic immunoregulatory function of nTregs is potentially less important than their local immune regulatory capacity and antigen specificity because they have no impact on the tolerogenic outcome.

However, translating antigen-specific nTreg therapy into a human setting is challenging because of the very low frequency of antigen-specific nTregs in the polyclonal T-cell repertoire [17,18]. In addition, the stability of the nTreg phenotype has been questioned in mice and humans, revealing a potential problem with antigen-specific therapies [19,20,21]. Although studies in mouse models with genetically marked Tregs have shown that post-thymic Tregs can convert back to effector T cells [22], analysis of Tregs from chronic inflammatory sites suggests a higher plasticity of the Treg phenotype [23,24]. As some trials aim to use donor-specific Tregs, loss of the Treg phenotype could be detrimental. Therefore, an alternative approach is to convert antigen-specific CD4^+^ Teffs to adopt a stable Treg phenotype and genotype by forcing ectopic expression of Foxp3 [25,26,27]. These converted Tregs (cTregs) are stable and function normally. Furthermore, additional FOXP3 transduction to stabilize human Tregs has been successful [28,29]. This has been used in clinical adoptive Treg trials (NCT05234190).

We addressed these issues by adoptively transferring alloantigen-specific (hereafter referred to as allospecific) *Foxp3*-transduced CD4^+^ T cells into a skin transplant model. Our findings demonstrate that *Foxp3*-transduced allospecific cTregs have long-term in vivo survival, can induce allospecific tolerance, and maintain general immune competence in transplanted mice. These transferred allospecific cTregs accumulated in the transplanted grafts. The importance of antigen-specific homing in the graft was demonstrated by the successful retransplantation of tolerized grafts in newly reconstituted hosts. We propose that allospecific Tregs have therapeutic potential for clinical use in SOT.

## 2. Materials and Methods

### 2.1. Mice

BALB/cJ and C57BL/6J mice were bred and maintained under pathogen-free conditions at the animal facility of the Hannover Medical School, Hannover, Germany. BALB/c-RAG1^−/−^ (c.129S7(B6)-Rag1^tm1Mom^/J) mice were purchased from Jackson Laboratory (Bar Harbor, ME, USA). F1 mice, which are the F1 generation from the cross between the BALB/c and C57Bl/6 strains, as well as BALB/c-FoxpGFP (C.Cg-Foxp3^tm2Tch^/J) mice, were bred in the MHH animal facility. All mouse strains expressed Thy1.2. Animal care, treatment, and experimental procedures were performed in accordance with institutional, state, and federal guidelines.

### 2.2. Generation of Allospecific Tregs

We used mixed lymphocyte reaction (MLR) in BALB/c mice and irradiated allogeneic C57Bl/6 splenocytes. Allospecific CD4^+^ T cells from BALB/c mice were activated and expressed CD25 and CD69. CD3^+^CD4^+^CD25^+^CD69^+^ alloantigen-specific activated T cells were sorted, transduced, and cultured. Because the transduction efficiency is between 30 and 70%, the Tregs are sorted to a high purity of >90% before adoptive transfer.

### 2.3. Retrovirus Production and Cell Transduction

Retroviral vector particles were generated as previously described [25,26,27]. In brief, the retroviral vectors pRSF91_IRES_Foxp3Thy1.1 and pRSF91_IRES_Thy1.1 were ectopically produced with the packaging vectors pEcoEnv_IRES_puro and pcDNA3. MLVg/p in 293T cells. Activated CD4^+^CD25^+^CD69^+^ T cells were transduced with Foxp3Thy1.1 or control Thy1.1 viral supernatant by spin infection with protaminesulfate and a multiplicity of infection (MOI) of five. Seventy-two hours after transduction, the transduced cTregs were isolated according to the expression of the congenic marker CD90.1 (Thy1.1) on their cell surface.

### 2.4. Suppression Assay

Stimulating splenocytes (C57Bl/6 or C3H) irradiated with 20 Gy and responding BALB/c-CD4^+^ T cells were cultured in a 96-well plate in RPMI medium with or without allogeneic or polyspecific Tregs. After 72 h, cells were pulsed with 1 µCi 3H-thymidine and frozen for 18 h. The uptake was measured using a scintillation counter. The data are presented as the mean cpm of triplicate assays. The proliferation of effector cells and their inhibition are presented as a stimulation index based on the proliferation of Teffs alone.

### 2.5. Adoptive Cell Transfer

Teffs (BALB/c-CD4^+^CD45RB^hi^ T cells) were diluted in PBS and injected intravenously into the tail vein on day −1 to reconstitute all BALB/c RAG1^−/−^ mice with or without Tregs.

### 2.6. Depleting Antibodies

To generate a niche in non-lymphopenic BALB/cJ mice, one milligram of Thy1.2 (30H12) was injected intraperitoneally five days before adoptive cell transfer.

### 2.7. Skin Transplantation

Skin transplantation was performed as previously described [30]. Briefly, mice were anesthetized, and full-thickness tail skin was grafted onto the lateral flank of the recipient mouse. The bandage was removed 10 days after transplantation. The grafts were observed every other day and considered rejected when no viable donor skin was present. Long-term tolerance was defined as 100 days. Grafts were removed from tolerant mice and regrafted onto the lateral flank of a new host.

### 2.8. ELISPOT Assay

IFN-γ ELISPOT kits have been purchased from Mabtech AB (Stockholm, Sweden). The kit contains anti-IFN-γ capture antibody, biotinylated anti-IFN-γ detection antibody, streptavidin alkaline phosphatase, and BCIP/NBT plus substrate. All other reagents used were of analytical grade. Multiscreen-IP plates (Merck Chemicals, Darmstadt, Germany) were coated with 100 µL/well of anti-IFN-γ capture antibody (15 µg/mL) in PBS and incubated at 4 °C overnight. Plates were washed with PBS and blocked with RPMI 1640 medium containing 10% fetal bovine serum (FBS) for 2 h at room temperature to prevent non-specific binding. After blocking, 2 × 10^5^ BALB/c cells per well were seeded into the plates in triplicate, as indicated. Cells were stimulated with 2 × 10^5^ stimulator cells as indicated for 24 h at 37 °C in a 5% CO_2_ incubator. Control wells contained cells in medium only. After incubation, the plates were washed with PBS containing 0.05% Tween-20 to remove cells and debris. Biotinylated anti-IFN-γ detection antibody (1 µg/mL) was added to each well and incubated for 2 h at room temperature. After washing, streptavidin-alkaline phosphatase was added, and the plates were incubated for 1 h at room temperature. Spots were developed by adding BCIP/NBT-plus substrate until optimal spot development was achieved (approximately 5–10 min), and the reaction was stopped by rinsing the plates with tap water. Spots were counted using an automated ELISPOT reader system.

### 2.9. Histology

Tolerant and rejected skin grafts were formalin-fixed and embedded in paraffin (FFPE) as previously described [31]. Five-micron sections were stained with hematoxylin and eosin (H&E) according to standard protocols. For immunohistochemistry, skin grafts were kept in 30% glucose for 6 h, fixed in formalin, and frozen in Tissue Tek. Cryosections (6–8 µm) were fixed in methanol/acetone and stained with CD4-Cy3 (RMCD4-2) (homemade), Thy1.1-AF488 (Ox-7, BD Biosciences, Heidelberg, Germany), Foxp3-AF647 (FJK-16s, Thermo Fisher Scientific, Dreieich, Germany) antibodies, and DAPI. Analysis was performed using an AxioCam MRm microscope (Zeiss, Jena, Germany) and the corresponding software, AxioVision Rel. 4.8 (Zeiss).

### 2.10. Data Analysis

The data were analyzed and visualized using FlowJo 10 software (BD Biosciences, Heidelberg, Germany), CytExpert 2 software (Beckman Coulter, Brea, CA, USA), and Prism 8 software (GraphPad, San Diego, CA, USA). Schematic workflows were generated using BioRender.com. Moreover, *p*-values were determined using two-way ANOVA and multiple comparison testing (Benjamini, Krieger, and Yekutieli post hoc test). * *p* < 0.05, ** *p* < 0.01, *** *p* < 0.001, **** *p* < 0.0001.

## 3. Results

### 3.1. Allospecific cTregs Prolonged Allograft Survival

The decisive parameter in the applicability of allospecific Tregs is their in vivo efficacy in transplantation models.

**Figure 1:**
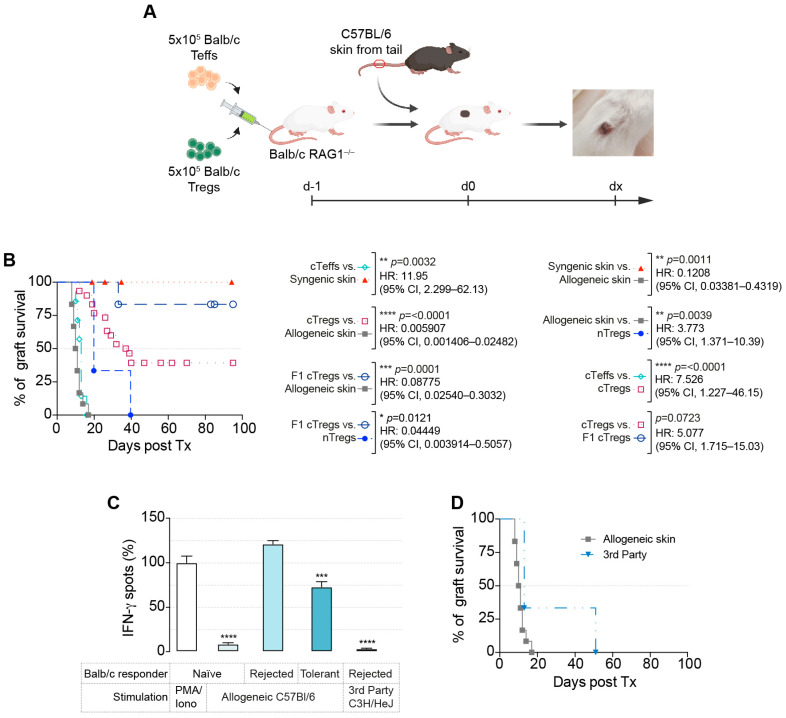
Allospecific cTregs prolonged allograft survival. (**A**) Schematic structure of skin grafting in the lymphopenic model. All BALB/c-RAG1^−/−^ recipients were reconstituted with 5 × 10^5^ BALB/c CD4^+^CD45RB^hi^ cells with or without additional Tregs on day −1. On day 0, C57Bl/6 skin was grafted onto the recipients. (**B**) Survival of syngeneic grafts (*n* = 4), allogeneic grafts without Tregs (*n* = 12), allogeneic grafts with nTregs (*n* = 3), Thy1.1 control vector-transduced cells (cTeffs; *n* = 7), allospecific cTregs (*n* = 30), and allospecific F1 cTregs (*n* = 6). (**C**) IFN-γ recall ELISPOT assays were performed with responder splenocytes from naïve, tolerant, or graft-rejected mice mixed with irradiated stimulator cells (cells from either naïve C57Bl/6J or naïve third-party C3H/HeJ mice). (**D**) Survival of third-party grafts (*n* = 3) and allogeneic grafts without Tregs (*n* = 12; from (**B**)).

Therefore, we established lymphopenic BALB/c RAG1^−/−^ recipients that were all reconstituted with 5 × 10^5^ BALB/c CD4^+^CD45RB^hi^ cells one day before skin transplantation (Figure 1A). Although syngeneic BALB/c skin was generally accepted, allogeneic C57Bl/6J skin transplants were rejected within 14 days (Figure 1B). The addition of 5 × 10^6^ polyspecific nTregs one day before allogeneic skin transplantation slightly delayed rejection (Figure 1B; *p* = 0.0039). Two different approaches were employed to test the effectiveness of allospecific cTregs. On the day before allogeneic skin transplantation, 5 × 10^5^ cTregs were adoptively transferred (Figure 1A,B). We found that 40% of the grafts showed long-term survival with allospecific cTregs (Figure 1B, cTregs, *p* < 0.0001). When allospecific F1 cTregs were used instead, more than 80% of the skin grafts survived (Figure 1B, F1 cTregs). While allospecific cTregs were stimulated directly by H2-k^d^ on C57Bl/6 cells, F1 cTregs were stimulated directly and indirectly by H2-kb before Foxp3 transduction [32]. Allospecific thy1.1-transduced Teffs had no significant effect on graft survival.

Additionally, an in vitro ELISPOT assay was performed to determine the specific capacities.

As a positive control, naïve BALB/cJ splenocytes were stimulated with PMA/ionomycin. This was set as 100% IFN-γ spots, and other results were normalized to this. While naïve T cells from BALB/cJ mice did not respond to allostimulation with irradiated C57Bl/6J splenocytes, primed T cells from BALB/c mice that rejected C57Bl/6J allografts showed a strong alloimmune response in the IFN-γ recall ELISPOT assay (Figure 1C). This IFN-γ response was much weaker when responder T cells were derived from tolerized BALB/cJ mice that had accepted C57Bl/6J skin allografts (*p* = 0.0029). In line with our hypothesis and third-party graft rejection (Figure 1D), even alloprimed T cells of BALB/cJ mice that rejected their C57Bl/6J graft did not respond and did not produce IFN-γ when stimulated with third-party C3H/HeJ splenocytes in vitro.

In summary, allogeneic cTregs are antigen-specific and can prolong graft survival.

### 3.2. Local Accumulation and Long-Term Survival of Tregs Restrain Graft Rejection

We aimed to determine whether local or systemic tolerance prevents allograft rejection.

**Figure 2:**
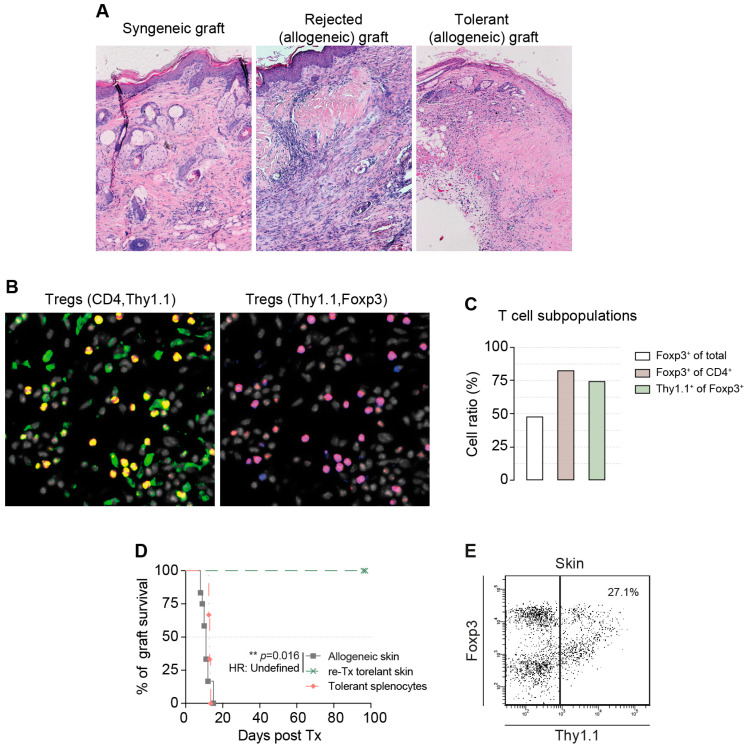
Local, but not systemic, tolerance restrained graft rejection. (**A**) H&E staining of explanted, tolerant syngeneic (**left**) or allogeneic skin with Tregs (**right**) or rejected allogeneic skin without adoptively transferred Tregs (**middle**). (**B**) IHC image of explanted, tolerant skin after 100 days (40×). Stainings for DAPI (white), CD4 (green), thy1.1 (red), and Foxp3 (blue) are shown. (**C**) The quantification of (**B**) is shown in these bar graphs. (**D**) Tolerant skin samples were regrafted onto reconstituted BALB/c-RAG1^−/−^ mice (re-Tx-tolerant skin, *n* = 2, *p* = 0.016) or reconstituted with tolerized splenocytes (*n* = 3, *p* = 0.38) before skin transplantation. (**E**) Flow cytometric analysis of T cells from inside the explanted graft.

The rejected allogeneic skin grafts showed massive lymphohistiocytes with severe necrosis in the skin sections (Figure 2A). There was pronounced acanthotic broadening of the epidermis and a dense orthohyperkeratosis. Hypergranulosis was observed in the epidermis. Hair follicles and sebaceous glands were absent in the fibrotic areas. Hair follicle fragments were surrounded by histiocytic, multinucleated foreign body giant cells. The upper and middle coria contained pigment-loaded macrophages, mostly melanophages. Surprisingly, explanted syngeneic skin grafts and tolerant allogeneic skin transplants from mice that received allogeneic Tregs showed mild lymphatic infiltration, acanthotic epidermal broadening, and orthohyperkeratosis (Figure 2A). Immunofluorescence analysis of tolerated skin revealed a high intradermal Treg/Teff ratio of almost 1:1 and a high Foxp3^+^ Treg percentage (80%) within the CD4^+^ T-cell subpopulation, demonstrating the tolerogenic status of the milieu (Figure 2B,C). Furthermore, we identified adoptively transferred allospecific cTregs by thy1.1 expression. Seventy-five percent of all locally accumulated Tregs were thy1.1^+^ and therefore allospecific cTreg in histological sections.

To demonstrate the critical importance of this locally induced tolerance, we explanted allogeneic C57Bl/6 skin grafts from long-term-tolerant mice. Tolerant skin was regrafted onto newly reconstituted BALB/c-RAG1^−/−^ mice. The regrafted skin was tolerated for another 100 days (Figure 2C; *p* = 0.016). Additionally, we performed another experiment with the same long-term tolerant donor mice by adoptively transferring tolerant splenocytes to reconstitute BALB/c-RAG1^−/−^ mice. The following day, allogeneic C57Bl/6 skin grafts were transplanted, but they were rejected within 14 days (Figure 2D). When explanted, tolerant skin grafts were analyzed by flow cytometry, no reconversion was observed. In line with the histological findings, more than 25% of all lymphocytes were transferred cTregs (Figure 2E).

In conclusion, the effect of systemic tolerance was limited in this model, whereas local immune tolerance prevented graft rejection.

### 3.3. Combination of Anti-Thy1.2 Antibodies with Rapamycin-Enabled Tolerance Induction by Treg Therapy

To generate a window of opportunity for tolerance induction with adoptive Treg therapy, we used anti-Thy1.2 antibodies and a short course of rapamycin.

**Figure 3:**
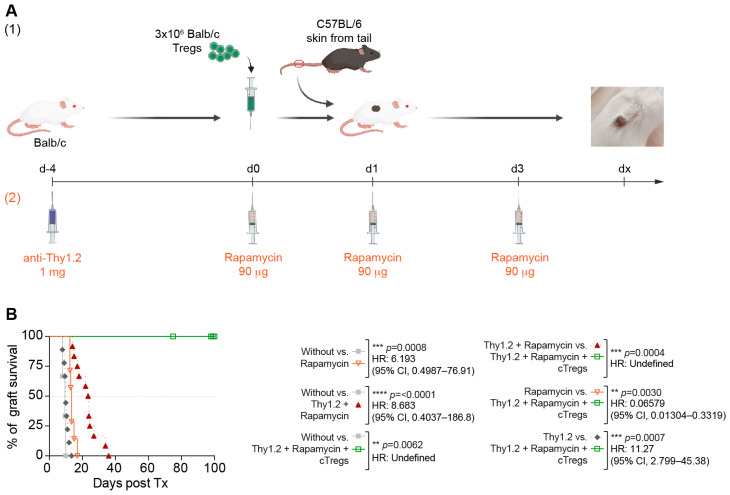
Allospecific cTregs induced long-term tolerance in an immunocompetent skin transplant model after niche generation. (**A**) Schematic representation of skin grafting in an immunocompetent mouse model. (**B**) Effects of rapamycin (*n* = 7), anti-Thy1.2 antibodies (*n* = 9), rapamycin with anti-Thy1.2 antibodies (*n* = 12), and cTregs with rapamycin and anti-Thy1.2 antibodies (*n* = 5) on allogeneic graft survival in the immunocompetent skin Tx model.

The thymocyte antigen Thy-2 is a classical T-cell marker. It is classically used for lymphocyte depletion because murine anti-thymocyte globulin (ATG) is no longer available [33]. Therefore, its action in murine models resembles the effect of the anti-CD52 antibody alemtuzumab and various ATGs used in clinical transplantation. Subsequently, we combined antiproliferative treatment with the mTOR inhibitor, rapamycin, and induction therapy. We intentionally inhibited mTOR to preferentially stop the proliferation of conventional T cells but only weakly affected that of Tregs [34,35]. Therefore, mTOR inhibition is routinely used by GMP facilities to prepare Treg populations for clinical trials (Figure 3A).

In the immunocompetent model, the combination of anti-Thy1.2 antibodies with rapamycin alone only slightly extended the allograft survival (Figure 3B), whereas the combination with a single application of cTregs led to long-term tolerance in all animals.

In conclusion, the combination of depletion therapy with anti-Thy1.2 antibodies and a short course of rapamycin enhanced adoptive Treg therapy and improved the translatability of this approach.

## 4. Discussion

Strategies that aim to minimize immunosuppression and induce tolerance via Tregs are currently at the forefront of clinical research on patients after SOT. Several trials in Asia [16] (NCT01624077), Europe (NCT04820270, NCT02166177, NCT02129881, NCT03867617, NCT01014234, NCT04924491, and NCT05234190), and North America (NCT02091232, NCT02188719, NCT01050764, and NCT03444064) are currently testing the safety and efficacy of adoptive transfer of immune regulatory cells [36]. However, we and others have shown that the mere transfer of Tregs after immunogenic transplantation (immunogenic strain mismatch and immunogenic tissue) is not sufficient to establish immune tolerance in rodent models [14,15,37]. Tang et al. showed that cyclophosphamide treatment shortly after transplantation can be used to deplete alloreactive T cells and enable tolerance induction by Treg transfer [15,38]. These murine data are supported by data from Japan in nonhuman primates and patients, showing that early weaning from immunosuppression is feasible with post-transplant cyclophosphamide and immune cell transfer [16,39,40]. However, post-transplant cyclophosphamide is not recommended after liver, lung, heart, or small bowel SOT, as postoperative infections still limit 3-month survival even if some studies have shown opposite results [40]. Therefore, we developed new strategies to facilitate adoptive Treg therapy using reagents commonly used in organ transplantation. To this end, we found that the combination of induction therapy, which can decrease alloreactive conventional T cells and increase the Treg/Teff ratio, with an antiproliferative strategy, which can ameliorate lymphopenia-driven expansion, creates a window of opportunity for successful adoptive Treg therapy. In fact, the combination of induction with a T cell-depleting antibody (the anti-Thy1.2 antibody) and rapamycin led to operational tolerance in all animals receiving cTregs. While the application of anti-Thy1.2 antibodies with rapamycin alone led to only a modest prolongation of graft survival, it generated a window of opportunity for adoptive Treg therapy, as all animals receiving Tregs developed lasting operational tolerance. This was accompanied by a significant increase in the Treg/Teff ratio in graft-draining lymph nodes and within the graft. In addition, we showed that transferred Tregs were enriched within the graft after transplantation. This is remarkable, as other studies have not found Tregs transferred two weeks after transplantation [15,41]. Our data also highlight the importance of the concomitant control of lymphopenia-driven T-cell proliferation, which would otherwise lead to a strong increase in alloreactive T cells. We used rapamycin because it has been shown that mTOR inhibition can suppress the proliferation of activated conventional T cells, with only minor effects on Tregs [35,42]. However, Reinke et al. demonstrated that calcineurin inhibitors (CNIs), such as cyclosporine, can be used in similar situations [43]. This is of clinical importance because CNIs are first-line immunosuppressants after organ transplantation. Similarly, Todo et al. treated Japanese patients with CNIs in combination with immunoregulatory cells after liver transplantation [16]. A recently published phase 1/2 trial (ARTEMIS; NCT02474199) confirmed these results [44].

It has been shown that antigen-specific Tregs are more potent than polyspecific Tregs in controlling unwanted immune responses [18,45]. We wanted to use polyspecific Tregs, if possible, because they are closest to the cells used in current clinical trials without chimeric antigen receptor technology. Therefore, we enriched the Tregs with this repertoire. Most experiments have shown that polyspecific Tregs are unsuitable for the treatment of autoimmune diseases because of the low number of tissue-specific Tregs in the nTreg repertoire [46]. In these experiments, lasting effects on autoimmunity were observed only with tissue-specific Tregs. However, the situation may be different for allotransplantation; we have recently shown that 8–12% of human nTregs are allospecific [18]. This is consistent with the results of Tang et al., who showed that 17% of nTregs in BALB/c and C3H mice were alloreactive [15]. Therefore, we used alloantigen-specific Tregs from the polyspecific repertoire to achieve operational tolerance. Similarly, Reinke and Tang have shown that fewer Tregs are needed if donor-specific Tregs are used, but tolerance is also feasible with polyspecific Tregs after transplantation [15,43].

To circumvent the stability issues caused by the transfer of nTregs [47] and ensure better follow-up of genetically marked cells, we used Foxp3-transduced CD4^+^ cells instead of nTregs. These cells showed stable Foxp3 expression in the periphery and the graft. Although cTregs do not have the exact epigenetic and expression profiles of nTregs, they have stable and lasting suppressive functions, as demonstrated by us and others [3,25,26,27,48,49]. To prevent the loss of the Treg phenotype by downregulating Foxp3, it may be useful to transduce nTregs before transfer to ensure stable suppressor function [28,29]. It is important to note that numerous trials have proven the safety of gene modification of differentiated T cells [50,51,52,53], and recent successful trials using T cells modified with chimeric antigen receptors (CARs) support this notion [54,55].

CAR modifications can circumvent the major problems of antigen specificity or allospecificity. This is a major problem in the clinical translation of Tregs into the clinic. Although safety has been demonstrated in most settings, its efficacy has suffered. This is due to the non-specificity of polyspecific Tregs. Antigen specificity via T-cell receptors will not be translatable beyond the experimental stage into the clinic because of technical difficulties and MHC restriction. Our approach cannot be implemented in clinical practice because of the lack of donor materials and the difficulty of implementing GMP regulations. Therefore, CAR-Tregs offer a promising alternative (NCT04817774 and NCT05234190) [12,56,57].

## 5. Conclusions

In summary, we showed that the success of adoptive Treg therapy after allotransplantation requires a window of opportunity. The use of currently available induction therapies in combination with a drug that limits lymphopenia-induced T-cell proliferation increases the Treg/Teff ratio, leading to marked Treg accumulation within the graft and inducing long-lasting operational tolerance. We believe that these results have important implications for future trials of adoptive Treg therapy after organ transplantation.

## Data Availability

The data presented in this study are available on request from the corresponding author.

## Abbreviations

ATG	anti-thymocyte globulin
GvHD	graft-versus-host disease
MLR	mixed lymphocyte reaction
MOI	multiplicity of infection
SOT	solid organ transplantation
Tregs	regulatory T cells
Teffs	effector T cells
cTregs	converted Tregs
nTregs	natural Tregs
